# Digital enzyme assay using attoliter droplet array

**DOI:** 10.1039/c8an01152d

**Published:** 2018-09-17

**Authors:** Takao Ono, Takanori Ichiki, Hiroyuki Noji

**Affiliations:** a Department of Applied Chemistry , Graduate School of Engineering , The University of Tokyo , Japan . Email: hnoji@appchem.t.u-tokyo.ac.jp; b Department of Materials Engineering , Graduate School of Engineering , The University of Tokyo , Japan; c ImPACT Program , Japan Science and Technology Agency , Saitama 332-0012 , Japan

## Abstract

Single-molecule digital enzyme assay using micron-sized droplet array is a promising analysis method to quantify biomolecules at extremely low concentrations. However, multiplex digital enzyme assays are still difficult to access because the best buffer conditions can vary largely among enzymes. In addition, the best conditions for flurogenic compounds to retain high quantum efficiency and to avoid leakage into the oil phase can be also very different. In this study, digital enzyme assay was performed using an array of nanometer-sized droplets of 200 aL volume, termed ‘nanocell’. Due to the small reaction volume, nanocell enhanced the accumulation rate of fluorescent products by a factor of 100 when compared with micron-sized reactors. Nanocell also enabled oil-free sealing of reactors: when flushed with an air flow, nanocell displayed water droplets under air, allowing enzymes to catalyze the reaction at the same rate as in oil-sealed reactors. Dual digital enzyme assay was also demonstrated using β-galactosidase and alkaline phosphatase (ALP) at pH 7.4, which is far from the optimum condition for ALP. Even under such a non-optimum condition, ALP molecules were successfully detected. Nanocell could largely expand the applicability of digital bioassay for enzymes under non-optimum conditions or enzymes of low turnover rate. The sealing of the reactor with air would also expand the applicability, allowing the use of fluorescent dyes that leak into oil.

## Abstract 2

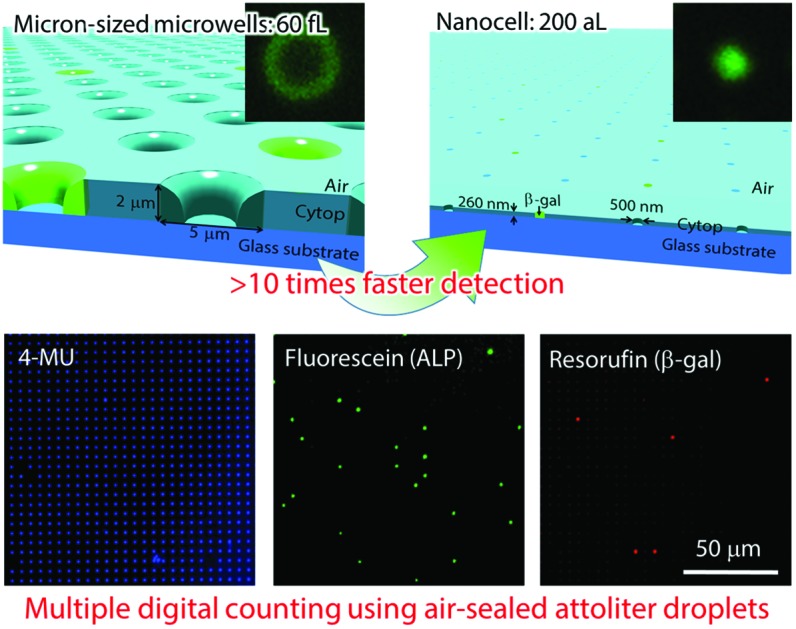
Attoliter-volume and air-sealed reactor array for fast and robust single-molecule enzyme assay.

## Introduction

When sensitivity of analytical methods is approaching single-molecule levels, biomolecules and their reactions exhibit their intrinsic nature of discreteness. Such quantized behavior has offered deep insights into the mechanisms of biochemical reactions.^1^–^9^ Due to the small size of biomolecules, micro/nanofabrication technology, which has been sophisticatedly developed for the semiconductor industry, provides useful tools for single-molecule analysis,^10^–^20^ allowing us to approach the discrete natures of biomolecules.

Single-molecule detection by microcompartmentalization is one of the most widely used applications of micro/nano devices for bioanalysis. A classic experiment for single-molecule detection with microreactors is DNA detection by PCR, where a DNA solution is partitioned into microwells so that zero, one or more DNA molecules are compartmentalized in the microwells. After PCR amplification, the number of DNA molecules in the original solution is determined from the counts of microwells giving a positive signal from amplicons.^21^ Due to the exponential amplification of PCR, single-molecule digital PCR does not require very small reactors, and, therefore, many formats of digital PCR have already been reported.^22^–^24^

On the other hand, single-molecule enzyme assay had to wait to be realized until regularly shaped micrometer-sized reactors became available. This is because output of enzymes, typically a signal from reaction product, increases linearly with time developed, and has to be highly condensed in a micrometer-sized reactor with a volume of a few femtoliters (1 fL = 10^–15^ L).^25^ In a typical protocol, enzymes are encapsulated in micron-sized reactors with a fluorogenic substrate that is hydrolyzed upon catalysis to generate a fluorescent reaction product. When the concentration of the enzyme is very low, most of the microreactors entrap none or only one molecule of enzyme stochastically. In such conditions, one can count the absolute number of entrapped enzymes as the number of the fluorescent reactors, after binarizing the fluorescent signal of reactors into positive and negative ones. Thus, these methods including digital PCR are referred as to “digital counting” or “digital bioassays”.^17^,^26^

In recent years, use of digital bioassays for protein detection has been expanding to detect non-enzyme molecules.^4^,^5^,^9^,^27^,^28^ One of the most actively studied methods is digital enzyme-linked immunosorbent assay (digital ELISA).^27^,^28^ In typical protocol for digital ELISA, target non-enzyme molecules are recognized by enzyme-conjugated antibodies and individually encapsulated into micron-sized reactors with fluorogenic substrates for the conjugated enzyme. The concentration of the target molecule is then determined by counting fluorescent reactors. Compared to conventional ELISA, digital ELISA markedly improves the limit of detection down to femto- or attomolar range. In theory, digital ELISA should not have a limitation in detection sensitivity as do other digital bioassays when having no false-positive signal. However, in reality, false-positive signal due to nonspecific adsorption of enzyme-conjugated antibody predominantly limits the detection sensitivity of digital ELISA.

Previously, we reported a dual-color digital bioassay to detect β-galactosidase (β-gal) and alkaline phosphatase (ALP).^29^ Multiplex digital assay has large potential impact on digital ELISA; it should enable not only parallel detection of multiple antigens, but also marked suppression of false positives. When epitopes on a single antigen molecule are recognized with antibodies conjugated with different enzymes that produce differently colored fluorogenic products, true positive reactors with multiple color signals should be well discriminated from false positives, most of which should show singly colored fluorescence. For example, in the case of dual-color assay, when 1% of reactors show false positive in each color channel due to nonspecific binding, the possibility to find reactors showing false positive in both channels should be 0.01% because nonspecific binding events are non-correlated stochastic processes. Although dual or multiple labelling with conjugates is less efficient for small antigens like peptides, it would be effective for ELISAs targeting large antigens like viruses or large proteins.

Some technical challenges still remain to be solved for realization of multi-color digital ELISA. The optimum buffer conditions vary among enzymes, and hence it is always a demanding task to find the best buffer condition for digital bioassay of enzymes. In addition, the fluorogenic compounds also limit the buffer conditions. One of the critical factors is pH that can largely affect the quantum efficiency of fluorescent products by modulating protonation state of the fluorescent dyes. Water–oil partition coefficient of fluorogenic substrate/product is also dependent on pH because electrical neutralization upon protonation/deprotonation facilitates leakage into the oil phase. Thus, even for dual-color digital bioassay, the buffer condition has to balance at least four limitations by a pair of enzymes and a pair of fluorogenic dyes. Therefore, there are demands not only for signal enhancement that enable digital assays of enzymes under non-optimum conditions but also for suppression of fluorogenic product leakage.

In this study, an array device of nanometer-sized droplet reactors was developed using nanoimprinting, and applied to digital counting. This novel device is named “nanocell”. The reaction volume of nanocell reactors (nano-reactors) is reduced to the attoliter level and concentration of reaction products increased more rapidly, so that digital counting of enzymes became more accessible even under non-optimum conditions for enzymes. In addition, it was demonstrated that nano-reactors can be sealed with air. Reaction substrates hardly diffuse from air-sealed nanocell. The signal increases rapidly so that evaporation of the droplets does not hamper the detection, unlike in the case of micron-sized reactors (μ-reactors).

## Experimental methods

### Materials

The D101S mutant of ALP from *Escherichia coli* and its fluorogenic substrate fluorescein diphosphate (FDP, Setareh Biotech, LLC (USA)) were generous gifts from Abbott Japan. β-Gal and its fluorogenic substrates, fluorescein di-β-d-galactopyranoside (FDG) and resorufin β-d-galactopyranoside (RGP), were purchased from Roche Diagnostics GmbH (Germany), Marker Gene Technologies, Inc. (USA) and Molecular Probes (USA), respectively. Resorufin and 4-methylumbelliferone (4-MU) were from Anaspec Inc. (USA) and Tokyo Chemical Industry Co. Ltd (Japan), respectively.

### Fabrication of nanocell

**Fig. 1:**
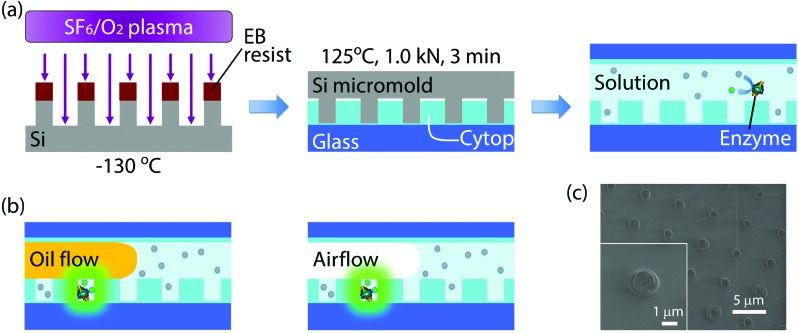
Schematics of the process for device fabrication and formation of attoliter droplet array. (a) Fabrication process for nanocell. Firstly, the micromold was cryo-etched at –130 °C. After EB resist removal, the micromold was employed for thermal nanoimprinting of nanocell. Nanocell was then filled with reaction solution containing enzymes and fluorogenic substrates. (b) Formation of attoliter-volume droplet array. The reactors were isolated by the flow of water-insoluble materials, fluorine oil or air. Once isolated, reactors containing enzyme molecules accumulated fluorescent products. (c) SEM image of nanocell. Inset shows a single attoliter-volume well.

Nanocell was fabricated by nanoimprinting and sealed by oil flow or air flow (Fig. 1(a) and (b)). Silicon micromold was patterned by electron beam (EB) lithography and etched using sulfur hexafluoride/oxygen mixture plasma at –130 °C in a cryogenic plasma etcher (Plasmalab 80plus, Oxford Instruments plc (UK)). The EB resist was then removed in acetone by ultrasonic cleaning. The device substrate was a glass coverslip (24 × 32 mm) cleaned in an oxygen plasma asher (PDC210, Yamato Material Co. Ltd (Japan)). The substrate was spin-coated with an amorphous perfluoropolymer (Cytop CTL-816AP, Asahi Glass Co. Ltd (Japan)) diluted to 5% in its solvent (CT-Solv180, Asahi Glass). After spin-coating, the solvent was evaporated on a hotplate. The evaporation conditions were as follows: the original temperature 50 °C was kept for 1 h, and the temperature was gradually raised to 180 °C for 1 h and finally kept at 180 °C for 1 h. The final thickness of the polymer film was 300 nm. Then the micromold was pressed against the substrate at 125 °C and 1.0 kN for 3 minutes using a nanoimprinter (NanoimPro Type 210, Nanonics Co. Ltd (Japan)).^30^ The micromold can be repeatedly used for nanoimprinting more than a hundred times. The resulting device, nanocell, had one million arrayed wells in a 5 mm square. Each well had a 500 nm radius and a 260 nm depth, and, therefore, was 200 aL in volume (Fig. 1(c)).

Nanocell was then assembled into a flow cell and filled with assay solution. To introduce aqueous solution inside nanocells, the device was cooled so that air inside nano-reactors was absorbed in aqueous solution and nanocell was filled with assay solution. As the oil flow or the air flow was conducted into the flow cell at a flow rate of 3 μl min^–1^, the solution was removed from the flow cell except for the inside of microwells (Fig. 1(b)). In some experiments of time-lapse imaging, fluorine oil (FOMBLIN YL-VAC 25/6, Solvay Solexis SpA (Italy)) was used instead of air flow for comparison with μ-reactors. In nanocell, droplets were surrounded by the perfluoropolymer and the oil or the air, not by glass, since the nanoimprinting does not penetrate the perfluoropolymer film.

### Fabrication of micron-sized microwells

The micron-sized microwells were fabricated similarly to previous reports.^31^ Briefly, a perfluoropolymer film was formed on a coverslip with 3 μm thickness. After patterning by photolithography, the perfluoropolymer film was etched in oxygen plasma using a conventional plasma etcher (RIE-10NR, SAMCO Inc. (Japan)). Finally, the photoresist was removed in acetone and ethanol. The resulting microwells had 2.5 μm radius and 3 μm depth, and, therefore, were 60 fL in volume.

### Enzyme assay

All assays were carried out in buffer containing 100 mM potassium phosphate at pH 7.4, 1 mM magnesium chloride and 0.1% v/v Tween20 (Sigma-Aldrich Co. LLC (USA)). For enzyme kinetic measurements and digital counting of enzyme, 50 μM FDG was used as substrate for β-gal and 200 μM resorufin was used for position identification of nano-reactors. In dual-color enzyme assay, 50 μM FDP and 200 μM RGP were used as substrates for 300 pM ALP and 300 pM β-gal, respectively, and 2.5 mM 4-MU was used for position identification. In this assay condition, product inhibition of ALP may be caused by phosphate buffer, although fluorescein product was successfully observed, due to the enhanced accumulation of product by nanocell as well as the tolerance of D101S mutant against product inhibition.^32^

### Fluorescence imaging and image analysis

Fluorescence images were obtained using an electron multiplying CCD camera (ImagEM Enhanced C9100-13, Hamamatsu Photonics K. K. (Japan)) and an inverted microscope (ECLIPSE Ti, Nikon Corp. (Japan)) equipped with 20× and 100× objective lenses (Plan Apo VC 20× (Nikon) and Plan Apo λ 100× oil (Nikon)). The 100× lens was used only for time-lapse imaging. Excitation and emission wavelengths were 470 nm and 535 ± 15 nm for fluorescein, 555 nm and 593 ± 20 nm for resorufin, and 395 nm and 480 ± 20 nm for 4-MU, respectively. For enzyme kinetic measurements and digital counting of enzyme, exposure time was 200 ms for fluorescein and 400 ms for resorufin. In dual-color enzyme assay, exposure time was 300 ms for fluorescein, 500 ms for resorufin and 100 ms for 4-MU. The fluorescence images were analyzed using ImageJ software (National Institutes of Health (USA)).

## Results and discussion

**Fig. 2:**
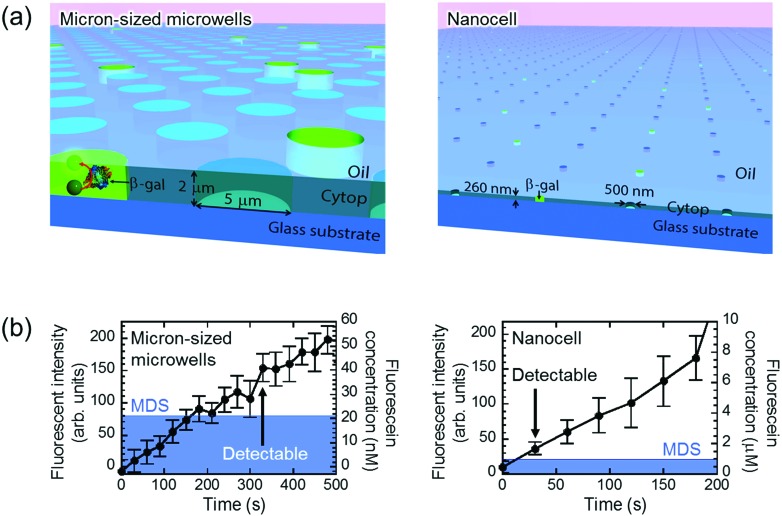
Detection of single-molecule enzyme in microwells sealed by oil flow. (a) Schematic images of the oil-sealed microwells. Since the oil prevented evaporation, both μ- and nano-reactors kept their shape and volume until detection of single-molecule enzyme. (b) Time course of the fluorescent intensity and fluorescein concentration in 60 fL and 200 aL reactors. Mean of background intensity, fluorescent intensity outside of the reactors, was subtracted from all data. Blue area represents MDS. β-Gal concentration was 5 pM in micron-sized microwells and 300 pM in nanocell. Error bars show one standard deviation of six data of micron-sized microwells and of eleven data of nanocell.

The principal advantage of nano-reactors in single-molecule digital enzymatic assay in comparison with μ-reactors is the rapid condensation of reaction product molecules, allowing swift detection. To confirm this point, we first conducted digital enzyme assay of β-gal with a fluorogenic substrate, FDG, that is hydrolyzed into galactose and fluorescein to give a fluorescence signal. Nano-reactors were sealed with fluorine oil after the reaction mixture of 50 μM FDG was loaded into the reactors. μ-Reactors were also tested for comparison purposes (Fig. 2(a)). In both reactors, fluorescence intensity increased almost linearly. μ-Reactors took over 300 s to reach a fluorescence signal above the minimum detectable signal (MDS) at 330 s. Herein, we define MDS as mean + 3SD of background noise (the fluorescent signal from outside of the reactors). In nano-reactors, the fluorescence signal increased more rapidly and reached above MDS within 30 s after encapsulation (Fig. 2(b)). The fluorescence signal increased 267 times faster in nano-reactors than in μ-reactors. The 267-fold acceleration is well consistent with the volume ratio of nano-reactors (200 aL) to μ-reactors (60 fL). The concentrations of the accumulated fluorescein in the reactors were determined with a reference data set of reactors filled with fluorescein solution at defined concentrations. The determined turnover rates of β-gal in both types of reactors essentially agreed with each other: 4.8 s^–1^ in nano-reactors and 5.2 s^–1^ in μ-reactors. Thus, β-gal molecules retain functionality in both reactor systems. Also these turnovers are in the same range as those on a bulk scale.^33^ With these turnovers, the amount of FDG substrate (approximately 6000 molecules in a single nano-reactor) is sufficient to keep constant reaction rate in the experimental time series. It is also worth mentioning that the noise level in nano-reactors was smaller than that in μ-reactors, because of the smaller size of nano-reactors that gives smaller background noise and smaller variance.

**Fig. 3:**
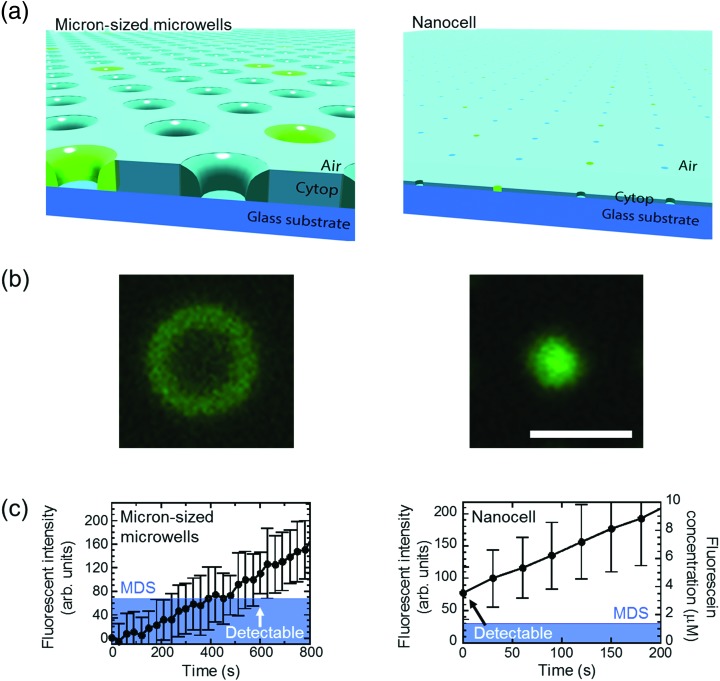
Detection of single-molecule enzyme in microwells sealed by air flow. (a) Schematic images of the air-sealed microwells. Due to evaporation, droplets in μ-reactors collapsed before detection of single-molecule enzyme. In contrast, attoliter droplets in nanocell kept their shape until detected. (b) Microscope images of air-sealed 60 fL μ-reactor (left) and 200 aL nano-reactor (right) with fluorescent products from single β-gal molecule. Scale bar: 5 μm. (c) Time course of the fluorescent intensity and fluorescein concentration in 60 fL and 200 aL reactors. The concentration in micron-sized microwells is not shown due to unstable droplet volume. Same as in Fig. 2, mean of background intensity was subtracted from all data and blue area represents MDS. β-Gal concentration was 10 pM in micron-sized microwells and 600 pM in nanocell. Error bars show one standard deviation of two data in micron-sized microwells and of six data in nanocell.

Next, we tested the possibility of forming droplet reactors under air flow with μ-reactors and nano-reactors (Fig. 3). In the case of μ-reactors, a large part of the buffer solution evaporated soon after excess buffer solution was flushed with air flow. The remaining solution was held along the circumference of the bottom of μ-reactors (Fig. 3(a)). Although the entrapped enzyme still catalyzed the fluorogenic reaction, the activity was evidently low. To reach above MDS, it took around 600 s, which is significantly longer than the detection time with oil-sealed μ-reactors (330 s) (left panels in Fig. 3). This is probably due to over-enrichment of buffer solutes such as inorganic salts. On the other hand, nano-reactors retained solution after air-sealing, probably due to larger surface interaction of aliquot with nano-reactors that would hold aliquot stably. Entrapped enzymes well catalyzed the reaction, and showed evident fluorescence (right panels in Fig. 3). Single-molecule enzyme detection was accelerated in nano-reactors the same as in oil-sealed nano-reactors. The rate constant of the enzyme turnover in air-sealed nano-reactors did not show a significant difference from that in oil-sealed nano-reactors.

**Fig. 4:**
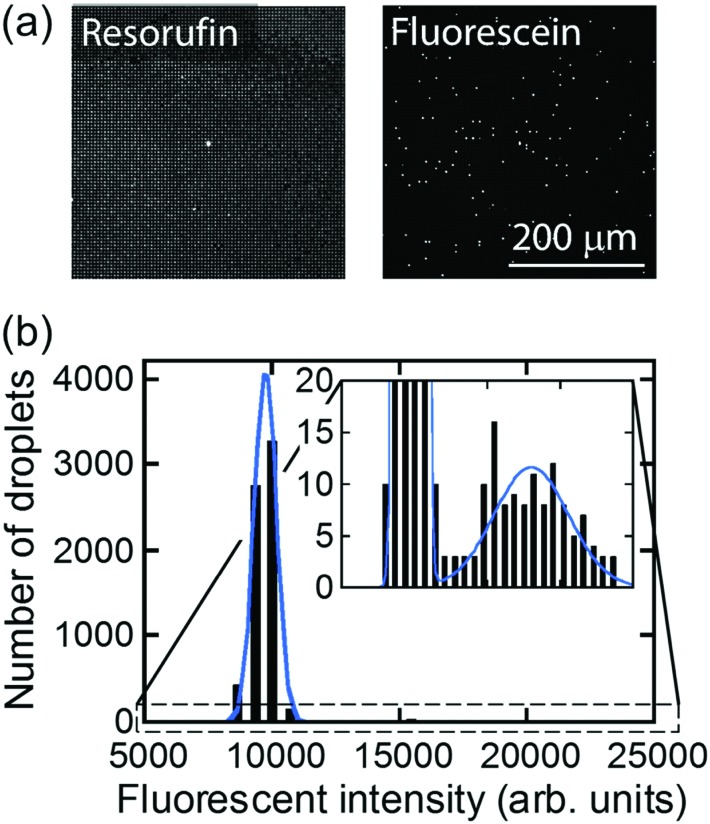
Distribution of fluorescent intensity of nano-reactors. (a) Fluorescence images taken at the same location. Resorufin was used for position identification of nano-reactors. Fluorescein showed the presence of β-gal. (b) Distribution of fluorescent intensity of fluorescein in the nano-reactors. The histogram was fitted by two Gaussian functions.

For further characterization of air-sealed nano-reactors, more quantitative analysis was conducted. Fig. 4(a) shows fluorescence images of air-sealed nano-reactors entrapping β-gal molecules with 50 μM FDG and 200 μM resorufin taken after about 3 min incubation. Because of the smaller size of the nano-reactors and refractive index matching of water and the perfluoropolymer,^34^ nano-reactors are barely visible under bright field imaging. Thereby, resorufin dye was introduced in the nano-reactors to visualize their positions. The fluorescent image of fluorescein shows that the reactors entrapping β-gal were randomly positioned among dark reactors as expected. In the histogram of the fluorescence intensity of fluorescein (Fig. 4(b)), two distinct peaks were clearly resolved: the left peak corresponds to the reactors without β-gal molecules and the right peak for the ones entrapping one or more β-gal molecules. This result indicates that β-gal molecules were randomly and individually dispersed in air-sealed reactors. Since the two peaks can be separated in the histogram, nanocell is able to be applied to digital counting of β-gal molecules by setting a threshold fluorescence signal of fluorescein. Reactors were identified as positive reactors when showing fluorescence intensity above MDS. The distribution of fluorescence intensity of positive reactors reflected a wide distribution of β-gal turnover. Consistent with coefficient of variation (CV) value reported elsewhere (around 42%),^35^ CV value of β-gal turnover was estimated as 31% from the data in Fig. 4(b).

Digital counting directly determines the expected value of the number of enzyme molecules per reactor volume, that is, enzyme concentration. As enzyme molecules were randomly distributed in reactors, the number of molecules in reactors should obey a Poisson distribution:1
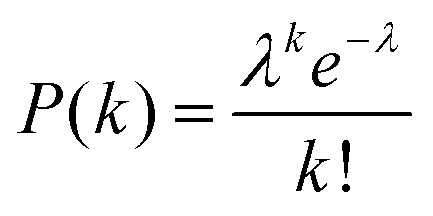



Here *P*(*k*) is the probability that *k* molecules are encapsulated in a reactor. *λ* is an average number of molecules per reactor that should be given by:

2 *λ* = *N*_A_*VC*

where *N*_A_ is Avogadro's number, *V* is the volume of a single reactor and *C* is the molar concentration of enzyme. The ratio of fluorescent reactors, *R*_FL_, and the enzyme concentration, *C*, are related as follows:

3 *R*_FL_ = 1 – *P*(0) = 1 – e^–*N*_*A*_*VC*^

**Fig. 5:**
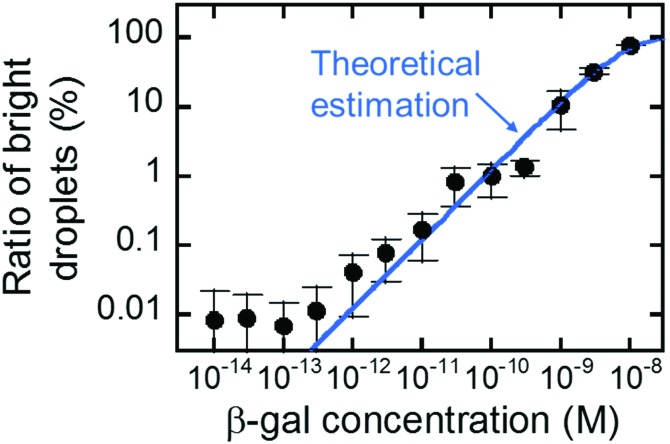
Digital counting of β-gal using nanocell. Blue line represents the theoretical curve from eqn (3). Error bars represent one standard deviation calculated from 4 to 34 data for each point.

Fig. 5 shows the concentration dependency of the ratio of the fluorescent reactors to the total reactors. The ratio increased monotonically with concentration and was almost consistent with the theoretical estimation from the enzyme concentration according to eqn (3) (blue line). This result shows that digital counting was performed using nanocell and it can quantitate the enzyme concentration from 100 fM to 10 nM. This coincidence also means the enzyme molecules were efficiently introduced into nano-reactors as in μ-reactors despite the larger surface-to-volume ratio. The dimension of nano-reactors (hundreds of nanometers) would be still too large to see exotic effects of extended nanospace.^36^ Less than 100 fM, the ratio becomes almost constant around 0.01%. It is false-positive. Statistically, we should observe some false-positive reactors, considering that background signal apparently obeys a Gaussian distribution that should contain exceptionally high background fluorescence reactors. This can explain one-fourth of the total false-positive reactors. The remaining three-fourths have to be attributed to impurities of unknown origin in the assay mixture or device materials.

**Fig. 6:**
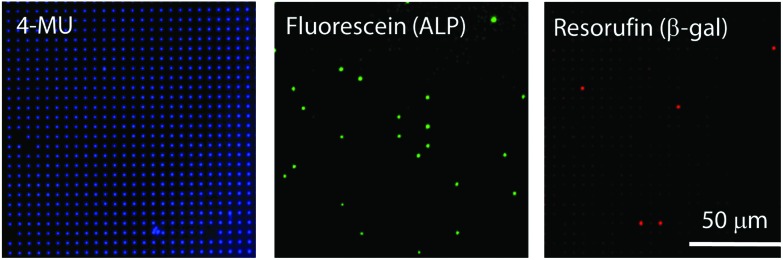
Dual-color enzyme assay using nanocell. The positions of nano-reactors were identified by 4-MU fluorescence. Fluorescein from FDP and resorufin from RGP show the presence of ALP and β-gal molecules, respectively. The nanocell encapsulated 2.5 mM 4-MU, 300 pM ALP, 50 μM FDP, 300 pM β-gal and 200 μM RGP in pH 7.4 potassium phosphate buffer. All images were obtained 16 min after encapsulation.

Finally, air-sealed nanocell was applied to dual-color digital enzymatic assay using ALP and β-gal with their fluorogenic substrates, FDP and RGP, which produce fluorescent dyes, fluorescein and resorufin, respectively. The optimum pH is 9.25 for ALP and 7.0 for β-gal.^29^,^37^ In a previous study,^29^ the dual-color assay was conducted at pH 8.25 to keep ALP activity high, although neutral pH is preferable for antigen–antibody reaction in digital ELISA. In this study, ALP and β-gal (300 pM each) were introduced together at pH 7.4 where ALP activity is 4-fold lower than at the optimum pH while β-gal retains almost maximum activity.^38^Fig. 6 shows the dual digital assay where ALP enzymes were entrapped at *λ* = 0.036 with β-gal molecules. Both ALP and β-gal activities were clearly observed. As expected, 3.5% of reactors showed ALP activity. Thus, it was confirmed that nanocell allows digital bioassay of enzymes even at non-optimum conditions due to the reduced reaction volume as well as perfect sealing with air. This enhances the robustness of single-molecule digital assay of enzymes, and expands the applicability of oncoming multi-color digital ELISA.

## Conclusions

Nanocell device was successfully developed for a swift and robust digital counting assay. Nanocell was fabricated by nanoimprinting and sealed by air flow to form a million 200 aL reactors, and allowed quantitation of enzyme concentration as low as 100 fM. It was also revealed that nanocell is able to detect a signal from a single enzyme molecule within 30 s, which is more than ten times faster than a previously reported micron-sized reactor system. Moreover, the dual-color enzyme assay was demonstrated for conditions far from the optimum for the enzyme, owing to the robustness of single-molecule detection using nanocell. This novel device may play a considerable role in single-molecule assays, not only dual-color digital ELISA, but also in analysis for reaction mechanisms of enzymes with low turnover rates that are difficult to access with conventional micron-sized reactors.

## Conflicts of interest

T. O. and H. N. of the University of Tokyo hold a patent, application number 2017-064794, on the method for forming microdroplets using microwells and air flow.
